# High-Resolution Magnetic Resonance Neurography at 7T: A Pilot Study of Hand Innervation

**DOI:** 10.3390/diagnostics14121230

**Published:** 2024-06-12

**Authors:** Pauline C. Guillemin, David Ferreira Branco, Yacine M’Rad, Loan Mattera, Orane Lorton, Gian Franco Piredda, Antoine Klauser, Roberto Martuzzi, Pierre-Alexandre Poletti, Rares Salomir, Sana Boudabbous

**Affiliations:** 1 Image Guided Interventions Laboratory (GR-949), Faculty of Medicine, University of Geneva, 1211 Geneva, Switzerland; mrad.yacine@gmail.com (Y.M.); orane.lorton@unige.ch (O.L.); rares.salomir@unige.ch (R.S.); sana.boudabbous@hcuge.ch (S.B.); 2 Radiology Department, University Hospitals of Geneva, 1205 Geneva, Switzerland; david.ferreirabranco@hcuge.ch (D.F.B.); pierre-alexandre.poletti@hcuge.ch (P.-A.P.); 3 Human Neuroscience Platform, Fondation Campus Biotech Geneva, 1202 Geneva, Switzerland; loan.mattera@fcbg.ch (L.M.); roberto.martuzzi@fcbg.ch (R.M.); 4 Advanced Clinical Imaging Technology, Siemens Healthineers International AG, 1015 Lausanne, Switzerland; gian_franco.piredda@siemens-healthineers.com (G.F.P.); antoine.delattre-klauser@siemens-healthineers.com (A.K.); 5 CIBM Center for Biomedical Imaging, 1202 Geneva, Switzerland

**Keywords:** ultra-high-field MRI, digital nerve mapping, Pacinian corpuscle, 3D curved planar reformation, automatic segmentation

## Abstract

The emergence of 7T clinical MRI technology has sparked our interest in its ability to discern the complex structures of the hand. Our primary objective was to assess the sensory and motor nerve structures of the hand, specifically nerves and Pacinian corpuscles, with the dual purpose of aiding diagnostic endeavors and supporting reconstructive surgical procedures. Ethical approval was obtained to carry out 7T MRI scans on a cohort of volunteers. Four volunteers assumed a prone position, with their hands (*N* = 8) positioned in a “superman” posture. To immobilize and maintain the hand in a strictly horizontal position, it was affixed to a plastic plate. Passive B0 shimming was implemented. Once high-resolution 3D images had been acquired using a multi-transmit head coil, advanced post-processing techniques were used to meticulously delineate the nerve fiber networks and mechanoreceptors. Across all participants, digital nerves were consistently located on the phalanges area, on average, between 2.5 and 3.5 mm beneath the skin, except within flexion folds where the nerve was approximately 1.8 mm from the surface. On the phalanges area, the mean distance from digital nerves to joints was approximately 1.5 mm. The nerves of the fingers were closer to the bone than to the surface of the skin. Furthermore, Pacinian corpuscles exhibited a notable clustering primarily within the metacarpal zone, situated on the palmar aspect. Our study yielded promising results, successfully reconstructing and meticulously describing the anatomy of nerve fibers spanning from the carpus to the digital nerve division, alongside the identification of Pacinian corpuscles, in four healthy volunteers (eight hands).

## 1. Introduction

The human hand is considered an “evolutionary marvel”. It has remarkable flexibility thanks to its fully opposable thumb. It also has an outstanding ability to feel and to touch. The human hand comprises an exceedingly complex nerve network essential for sensitivity, motricity, and proprioception, and the nerves of the hand serve as conductors and receptors of mechanical pressure.

The single nerve fiber called axon is the functional unit of peripheral nerve. It’s surrounded by a layer of connective tissue called endoneurium; several axons constitute the fascicle and are surrounded by the perineurium. The epineurium separates fascicles [1]. The endings of nerves are called Pacinian corpuscles (PCs). A histological description of Pacinian corpuscles by Stark et al. [2] showed that these structures are located in the subcutaneous tissue as clusters in the vicinity of the common digital nerve and its branches, randomly orientated with respect to the nerves and the skin, and most numerous in the metacarpophalangeal area and first phalanx [2]. These PC structures provide sensibility and recovery correlated with the re-innervation of these structures. A range of 192 to 424 was reported as clusters, adjacent to nerves branches, in contrast to monkey hands [2].

However, visualizing this complex network proves challenging. The motor innervation of the hand has a complex additional variation, with many classification and systems used and a relevant impact on clinical implications such as nerve iatrogenic injuries. This nerve network is developed in a relatively old anatomic atlas without reference to a recognized and interactive imaging modality to show this relevant information for juniors and fellows. The use of ultrasound can assist in the detection of patients with a bifid median nerve or persistent median artery (PMA), which are populations more prone to harboring median nerve variations [3]. Despite its usefulness, the efficacy of ultrasound in visualizing the intricate details of the nerve network is limited. Ultrasound imaging is often hindered by several factors including operator dependency and the lack of a spatial reference frame, which can result in variability in image acquisition and interpretation. Additionally, the limited field of view restricts the ability to capture comprehensive anatomical details. The visualization of deep structures is also challenging due to poor acoustic penetration and resolution, which can obscure the detailed anatomy of smaller or deeper nerves.

Furthermore, conventional MRI performed at lower field strengths provides only limited insight, particularly when tasked with delineating complex multiplanar nerve pathways, thereby necessitating the adoption of labor-intensive three-dimensional imaging methodologies. Multiplanar nerve pathways require three-dimensional (3D) examination, which is a time-consuming imaging process. Although 3D computer models were developed for the human hand with exquisite detail, their spatial resolution and contrast for small structures remain low, as seen in the model created by Gehrmann et al. [4], which used CT imaging and described only large nerves like the median nerve. This technical development would benefit from a better resolution of nerve structures for anatomy teaching, surgical planning, and pain control.

Even though 3T MRI assesses proximal nerve structures (e.g., brachial and lumbosacral nerve plexi), it is limited for distal nerves and has not yet been described in the literature for finer nerves. This includes preoperative and post-traumatic assessments [5], the identification of partial fascicular nerves, and the differential diagnosis of an alleged somatoform disorder [6]. To this end, ultra-high-field MRI with three-dimensional (3D) anatomical sequences presents unparalleled capabilities to visualize anatomical details within the musculoskeletal system [7,8], in particular hand anatomy [9]. The inherent enhancement in signal-to-noise ratio (SNR) facilitates image acquisition and the 7T scanner can result in higher resolution, thinner slices, and faster imaging, unveiling specific structures invisible at a lower field strength [10,11,12].

This is of interest for the hand and wrist due to the small size of the anatomic structures. While these investigations mainly centered on data from healthy volunteers, without specific emphasis on nerve imaging, comparative studies across various field strengths demonstrate that 7T MRI allows the accurate delineation of nerves, Pacinian corpuscles, ligaments, and vessels, thereby promising an improved diagnosis of pathological conditions [10,13]. Correlation studies between anatomical findings and MRI abnormalities conducted on cadaveric extremities underscore a robust alignment between images and histological observations [14]. Furthermore, a study employing pre-clinical 11.7T MRI clarified the identification of tendon insertions, pulley systems, and neurovascular bundles within MR images of the interphalangeal joint [15].

Nevertheless, imaging peripheral nerves at 7T ex vivo remains challenging due to limited contrast and local field inhomogeneity. Moreover, in vivo, the scarce availability of suitable 7T transmit–receive coils is an obstacle to nerve imaging [16]. With regard to the Pacinian corpuscle detection study by Germann C and al. [11], using a 28-element-receive, single-transmit coil, the study provides valuable information on the distribution and arrangement of Pacinian corpuscles; however, these results are based on only two hands and there was no explicit tracking of nerves in the fingers.

We aim to assess the nerve structures of the hand in healthy adults using ultra-high-field MR imaging to determine the distality of collateral nerves and Pacinian corpuscle placement, which provides significant benefits in the context of microsurgery involving nerves. Originally, a multi-transmit coil was used to achieve a more homogeneous signal. The challenge of this feasibility study lies in the difficulty of acquiring MRI images and managing post-processing.

## 2. Materials and Methods

### 2.1. Volunteers and Setup

Four volunteers were recruited for bilateral hand imaging during a study period of 14 weeks. Status details are presented in Table 1.

Prior to the MRI examination, written informed consent was obtained from all participants. Exclusion criteria for this study mirrored those for standard MRI examinations and included conditions such as claustrophobia (due to the confined space of the MRI tunnel), incompatibility with MRI equipment (due to risks of image interference or danger to the patient), and a history of hand trauma or surgery (as we preferred healthy volunteers for this initial study). During imaging, volunteers assumed a prone position, with their hands positioned in a “superman” posture. To immobilize and maintain the hand in a strictly horizontal position, it was affixed to a plastic plate. Additionally, the hand was encased in a 2-L silicone balloon filled with commercial grade polypropylene (PP) pellets of an average diameter of 2 mm. PP is an electrical insulator and a weak diamagnetic material with a bulk susceptibility of −9.55 ppm [17], very close to the water susceptibility of −9.03 ppm. Looking at the infra-millimeter scale, the susceptibility distribution of the pellet arrangement, hosting air-filled interstices, is inhomogeneous. However, at a macroscopic scale of several centimeters, as is relevant for the current FOV and anatomy, its average susceptibility is close to that of biological tissue, largely improving the macroscopic tissue-to-air susceptibility contrast. Therefore, this home-designed setup improves passive B0 shimming by smoothing out the macroscopic distribution of the overall magnetic susceptibility of the objects (hand and surrounding pellets). Unlike aqueous gels, PP does not produce an MR signal that could interfere with the radiological readings of the anatomy. MR sequence acquisition took about 30 min and the total duration of the session for one hand was 45 min, taking into consideration the installation of the volunteer and the planning sequences. The volunteer could stop the acquisition at any time.

### 2.2. MR Imaging

Data were acquired using a 7T whole body MR scanner (MAGNETOM Terra.X, Siemens Healthineers, Forchheim, Germany), equipped with a head coil with 32 receive elements and 8 transmit channels with independent control. Three optimized sequences were performed: a multi-slice 2D proton density (PD) turbo spin echo (TSE) fat-saturated (FatSat) sequence (TSE, TE: 22 ms, TR: 14480 ms, flip angle: 130°, R: 0.2 × 0.2 × 1.5 mm^3^, bandwidth: 300 Hz/px, TA:10:23 min, Turbo Factor: 7); a second multi-slice 2D PD TSE sequence without FatSat (TSE, TE: 22 ms, TR: 14480 ms, flip angle: 130°, R:0.2 × 0.2 × 1.5 mm^3^, BandWidth: 300 Hz/px, TA:10:23 min, Turbo Factor: 7); and a 3D T2 DESS sequence (TE: 5.22 ms, TR: 11.06 ms, flip angle: 18°, BandWidth: 376 Hz/px, TA:10:23 min, R: 0.4 × 0.4 × 0.5 mm^3^, B1 shim volume selective, spectral selective binomial water excitation).

### 2.3. Post-Processing

In a first step the quality of the images was independently analyzed by two radiologists on the 3D DESS sequences, using a numerical scale from 0 to 2 (0: insufficient clinical quality; 1: diagnostic quality; 2: very good quality). The hand was divided into six analysis zones, palmar and dorsal sides of the metacarpophalangeal-proximal zone (MCP zone), the interphalangeal proximal zone (IPP zone), and the interphalangeal distal zone (IDP zone).

For each study participant, a meticulous approach was undertaken to visually track the four nerves of each finger in the serial axial planes, using Osirix’s advanced 3D Curved MPR function (CMPR, Osirix Dicom viewer, Geneva, Switzerland), on the T2 DESS sequence. The key purpose of this specialized feature is to rectify the inherent tortuosity of nerve structures, thereby facilitating precise visualization and analysis. Through this technique, a Curved Planar Reformation (CPR) image of each individual nerve within a single coronal slice was generated, effectively transforming its convoluted path into a more linear-like structure. Subsequently, a comprehensive set of measurements was undertaken to characterize each nerve, encompassing its dimensions from the base of the first metacarpal to the head of the distal phalanx, its proximity to the skin and adjacent joints, and its thickness. These measurements were meticulously referenced to ensure accuracy and consistency throughout the analysis.

Furthermore, in a parallel endeavor, Pacinian corpuscles were targeted for segmentation in three dimensions (3D) on the T2 DESS sequence using the versatile software tool 3DSlicer (v5.0.3, https://www.slicer.org/ (accessed on 10 March 2024)) [18]. This segmentation process entailed the optimized selection of a precise threshold tailored to the specific sequence parameters used for nerve tracking, with results tailored to the unique anatomical features of each volunteer. Subsequently, leveraging a dedicated Matlab script developed in-house (R2021a, The Mathworks, Natick, MC, USA), an automated quantification of Pacinian corpuscle distribution was executed with precision and efficiency. This automated approach not only speeded up the analysis process but also minimized the potential for subjective biases, thereby enhancing the reliability and reproducibility of the results. Through these meticulously procedures, a comprehensive dataset capturing the intricate morphology and distribution of both nerve structures and Pacinian corpuscles was assembled.

## 3. Results

### 3.1. Quality Assurance

**Figure 1:**
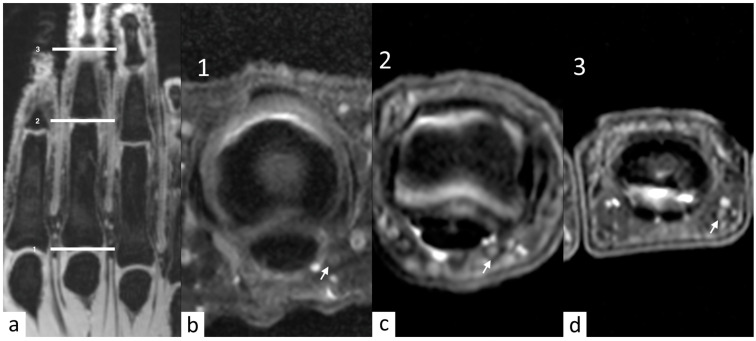
(**a**) Nerve quality analysis on three levels on axial DP (metacarpophalangeal joint, proximal interphalangeal joint, and distal interphalangeal joint). (**b**–**d**) Numbers 1, 2, and 3 show an example of a digital ulnar nerve which is well visible, with an intermediate signal intensity and the relationships with surroundings structures (e.g., subcutaneous fat and blood vessels) (arrows).

The analysis of the eight hands showed that image quality was overall very satisfactory across all zones (see Table 2), the digital ulnar nerve was clearly visible (Figure 1), and radiologists scores were in good agreement with no zero scores among 96 assignments.

**Figure 2:**
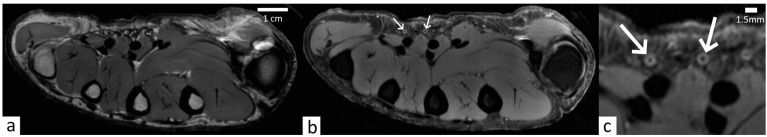
(**a**,**b**) Two-dimensional PD TSE and PD TSE FatSat MRI images in the axial plane of volunteer 3’s right hand. (**c**) Close up view of (**b**). Indicated by white arrows are vascular walls of small arteries (outer diameter = 1.3 mm), which are sharply depicted on the FatSat sequence and cannot be identified on the non-FatSat one.

The proton density (PD) sequences demonstrated outstanding uniformity in fat suppression, as illustrated in Figure 2. We employed this sequence as quality assurance of the 7T MRI system case-specific performance, to ensure confidence in subsequent findings. It is worth noting that the performance of B0 and B1 shimming is crucial for 7T MRI [19]. Besides the built-in active shimming of the MRI system, we also validated, for the current purpose, our home-designed passive shimming technique based on these results.

No motion or flow-related artefacts were visually detected on any of the images.

### 3.2. Nerve Description

**Figure 3:**
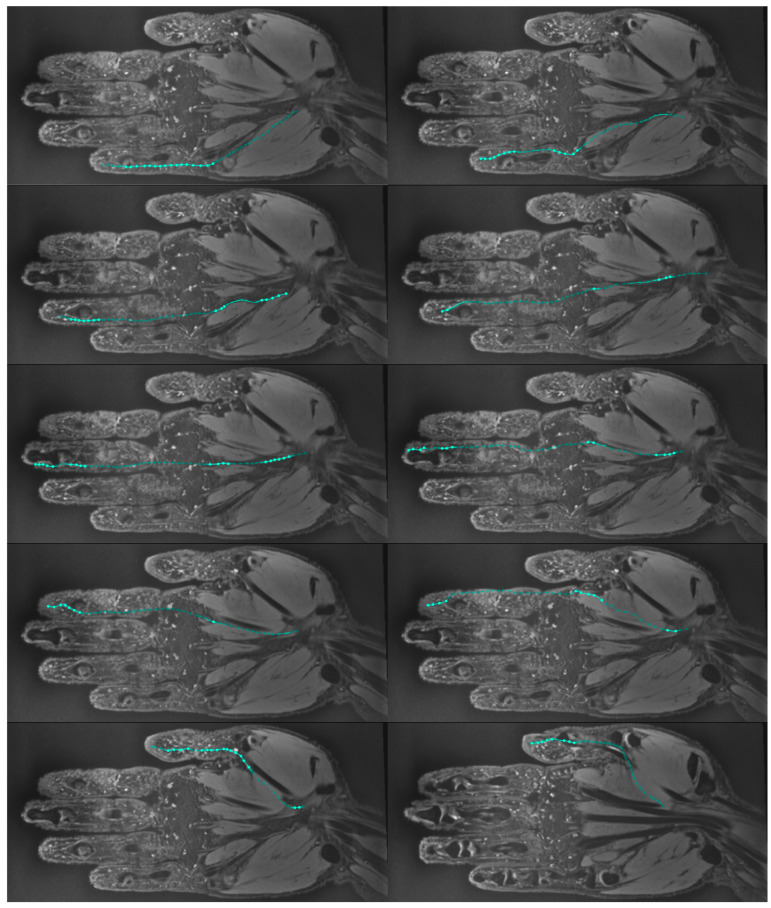
T2 DESS 3D MRI images of volunteer 3’s left hand in the coronal plane with palmar nerve paths, manually tracked with the 3D curved planar reformation function, as provided by OSirix (see blue lines and dotted landmarks).

Through the utilization of the T2 DESS sequence, a precise reconstruction of all digital nerves was achieved, as illustrated in Figure 3, further enhancing anatomical comprehension. For the eight scanned hands, 80 palmar nerves were reconstructed in 3D.

**Figure 4:**
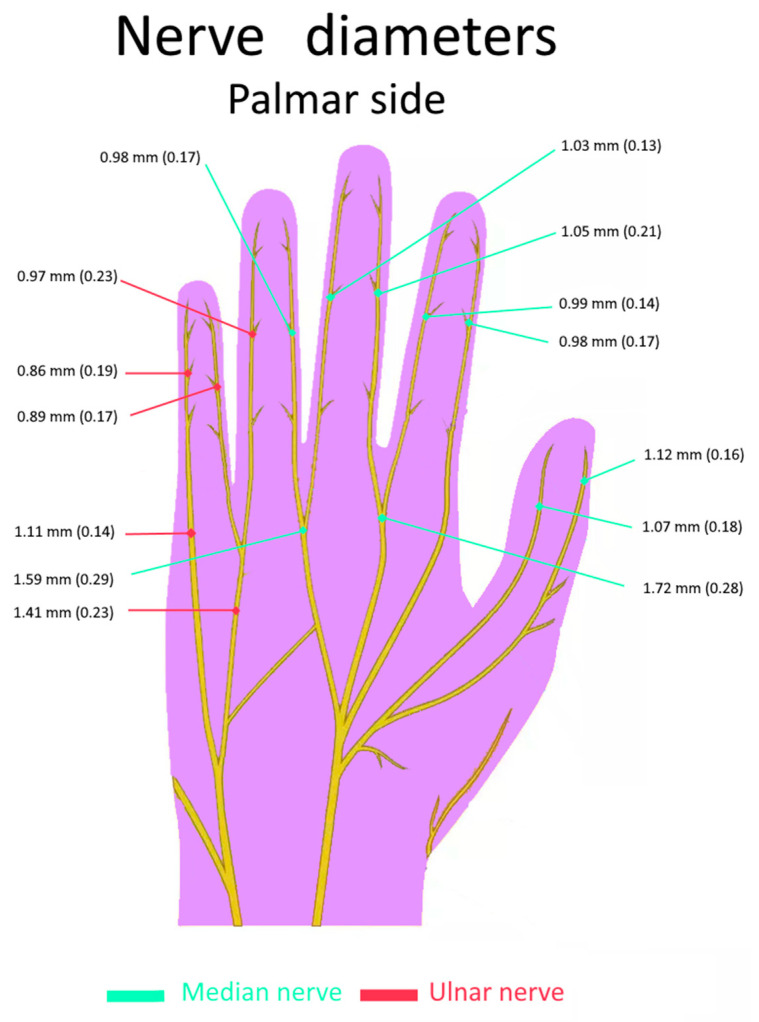
Scheme of hand nerve diameters in our population of eight hands. The average diameters and the respective standard deviations are provided for the branches of median and ulnar nerves.

Notably, segmentation was greatly facilitated by distinct variations in signal intensities: bones exhibited an average intensity of 34.5, muscles 62.8, nerves 100.78, and Pacinian corpuscles 178. This range clearly overweighted the residual spatial inhomogeneities in the MR signal, which were mainly due to the sensitivity profile of the multi-channel transmit–receive coil. Additionally, the T2 DESS sequence provided a sharp demarcation of nerve fascicles, with a clear identification of the perineurium of each fascicle. Table 3 presents comprehensive records of distances to joints and skin for ten palmar nerves of the right hand of a volunteer, and Figure 4 presents the average diameters of each nerve in the hand.

Across all participants, digital nerves were consistently located between 2.5 and 3.5 mm beneath the skin, except within flexion folds where the nerve was approximately 1.8 mm from the surface. In the phalanges area, the mean distance from digital nerves to joints was approximately 1.5 mm. The nerves of the fingers are closer to the bone than to the surface of the skin, but this situation is reversed in the metacarpal region (palm area). Indeed, in the palm area, the nerve was approximately 5.5 mm from the surface and 7.5 mm from the metacarpals.

### 3.3. Pacini Description

**Figure 5:**
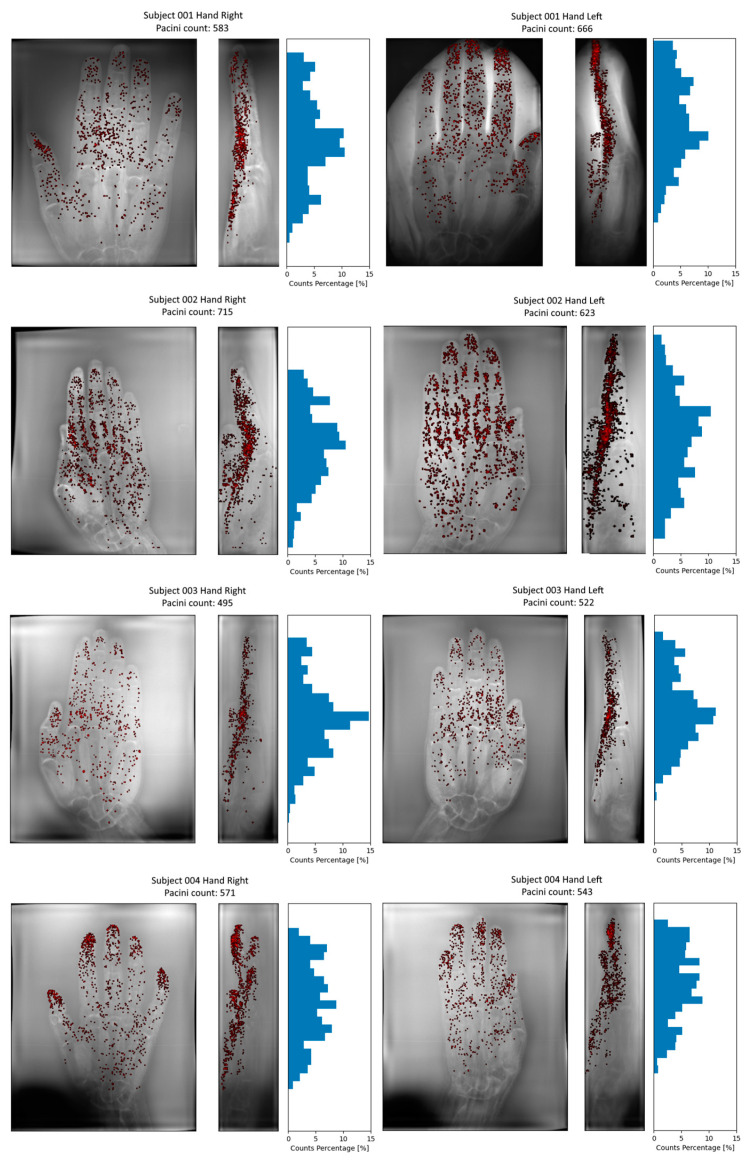
Three-dimensional segmentation of Pacinian corpuscles using 3DSlicer for the eight hands, with their respective proximal-to-distal histogram of spatial distribution.

The Pacinian corpuscles, as segmented across eight cases, exhibited a notable clustering primarily within the metacarpal zone, situated on the palmar aspect and located deep within the dermis of the skin, as shown in Figure 5.

The MRI aspect of Pacinian corpuscles on our 3D DESS high resolution images matched the characteristics of normal Pacinian corpuscles [20]: subcutaneous nodules separate from vessels, round or oval, 1–5 mm in diameter, concentrated in subcutaneous fat of the palms, with a highly specific “bunch of grapes” aspect alongside the digital nerves, with thin nerve extensions connecting them to the digital nerve being frequently observed. We also took advantage of the excellent fat signal suppression performance of our setup, which enabled a clear visualization of Pacinian borders.

Moreover, the abundance of Pacinian corpuscles appears to be markedly influenced by individual subject characteristics: for instance, volunteer two exhibited 623 corpuscles for the right hand and 715 for the left, while volunteer three demonstrated 495 corpuscles for the right hand and 522 for the left.

## 4. Discussion

Our investigation demonstrates the potential of high-resolution 7T MRI imaging to elucidate the intricate architecture of nerve fascicles within the hand. Through meticulous reconstruction, we successfully delineated the anatomy of nerve fibers spanning from the carpus to the digital nerve division, as well as the distribution of Pacinian corpuscles. This advancement holds significant promise in the realm of microstructural imaging of traumatic lesions within the peripheral nervous system, offering improvements in both anatomical visualization and tractographic analysis.

The potential clinical implications of our methodology are multifaceted. Firstly, our detailed imaging description of the nerve anatomy and termination in the human hand brings new insights into peripheral nerve morphology. By providing a comprehensive visualization of nerve structures, our study suggests new modalities for the refinement of anatomical knowledge in this domain, which is fundamental for various medical disciplines, including surgery, neurology, and rehabilitation. In the longer term, such imaging data may serve as a valuable resource for surgical planning, offering surgeons a more nuanced understanding of the spatial relationships between nerves and surrounding tissues. With enhanced pre-surgical diagnosis, surgeons can approach nerve-related procedures with greater confidence, minimizing the risk of iatrogenic injuries and optimizing patient outcomes. Additionally, our findings could pave the way for the development of tailored surgical interventions, where precise anatomical knowledge guides the selection of surgical approaches and techniques.

Moreover, our study highlights the utility of advanced imaging techniques, such as 7T MRI, in addressing clinical challenges associated with peripheral nerve pathology. For instance, through the visualization of microstructural alterations indicative of traumatic lesions, 7T MRI has the potential to support the diagnosis and management of peripheral nerve injuries. This includes the detection of subtle abnormalities, such as partial fascicular injuries, which may have significant implications for patient prognosis and treatment planning.

The enhanced signal-to-noise ratio (SNR) and spatial resolution at 7T MRI enable the visualization of minute anatomical details that are otherwise indiscernible. For example, a study by Welsch et al. [21] involving 10 healthy volunteers revealed that 7T MRI provided an enhanced evaluation of cartilage, offering higher spatial resolution, an improved contrast-to-noise ratio (CNR), and potentially reduced acquisition time. Similarly, Juras et al. [22] conducted a study with 10 subjects, showing substantial benefits from the ultra-high-field MR imaging of ankles with routine clinical sequences at 7T compared to 3T. Higher SNR and CNR at ultra-high-field MR scanners are useful in clinical practice for ankle imaging. These studies underscore the utility of 7T MRI in musculoskeletal imaging, highlighting its ability to offer detailed insights into various anatomical structures. In comparison to other studies utilizing 7T MRI within the musculoskeletal system, our findings align with the broader application of ultra-high-field imaging in this domain.

While our study demonstrates the potential of high-resolution 7T MRI imaging in elucidating the intricate architecture of nerve fascicles within the hand, it is essential to acknowledge several limitations. Firstly, our study comprised a relatively small sample size of four volunteers, which may restrict the generalizability of our findings to larger populations. Additionally, it is noteworthy that we did not provide histologic confirmation that the structures identified using MRI indeed correspond to Pacinian corpuscles. Addressing these limitations comprehensively is paramount for establishing the utility and reliability of high-resolution 7T MRI imaging in the assessment of peripheral nerve anatomy and pathology. The lack of a dedicated coil for hand anatomy was alleviated by using the “superman” prone posture of the subject, which, however, is susceptible to fatigue and accidental motion during longer immobilization periods.

## 5. Conclusions

In conclusion, our investigation highlights the transformative impact of high-resolution 7T MRI imaging on advancing our understanding of peripheral nerve anatomy and pathology. By providing detailed insights into nerve morphology and distribution, our study offers a valuable resource for both research and anatomy courses. As we continue to refine and expand upon these imaging methodologies and carry out further studies with more samples, we anticipate further advancements in the diagnosis and treatment of peripheral nerve disorders with the creation of an atlas with MRI images.

## Figures and Tables

**Table 1:** Volunteer information.

Volunteers	Age	Gender	Dominant Hand	Manual Work
1	34	M	Right	No
2	49	M	Right	No
3	31	M	Right	No
4	57	F	Right	Yes

**Table 2:** Mean score given by two radiologists for image quality.

	MCP Zone	IPP Zone	IPD Zone
Dorsal side	2	1.2	1.3
Palmar side	2	1.9	1.9

**Table 3:** Detailed characteristics of the palmar nerves of volunteer 2’s right hand. The measurement is taken between the point of the nerve furthest from the skin or joint and the point of the nerve closest to it (usually at the junctions). N/A: for the thumb which had only two phalanges.

		Distance from Nerve to Skin (mm)				Distance from Nerve to Joint (mm)			
Finger	Nerve Laterality	Metacarpals	Proximal	Middle	Distal	Metacarpals	Proximal	Middle	Distal
I	Left	3.9–6.9	2.4–3.9	N/A	2.2–4.3	8.7–10.0	1.8–3.3	N/A	1.2–1.6
	Right	4.3–6.6	2.4–4.1	N/A	2.0–4.4	7.6–11.3	2.4–3.5	N/A	1.1–1.5
II	Left	4.8–6.8	2.5–3.9	2.0–3.9	1.9–3.3	6.6–9.8	1.8–2.8	1.6–2.0	1.3–1.7
	Right	4.4–6.9	2.9–4.4	2.0–3.4	1.9–3.2	6.5–9.4	1.2–2.5	1.9–2.2	1.1–1.5
III	Left	4.5–7.3	2.5–4.9	1.9–3.2	2.0–3.3	6.9–8.6	1.1–2.3	1.9–2.4	1.1–1.6
	Right	3.9–6.5	2.4–4.2	1.9–4.2	1.8–2.9	6.4–9.2	2.2–3.4	2.0–2.8	1.3–1.6
IV	Left	4.1–7.9	2.3–4.2	1.8–2.8	1.8–2.0	5.2–6.8	1.4–2.7	1.2–2.0	1.2–1.9
	Right	4.3–5.8	2.5–3.9	1.8–2.4	1.5–2.4	5.2–8.9	1.4–2.9	1.2–2.7	1.2–1.7
V	Left	3.5–5.6	2.8–4.7	1.7–2.9	1.5–2.4	4.9–5.6	1.2–2.3	1.3–1.9	1.2–1.7
	Right	3.8–5.9	2.5–4.6	1.8–2.8	1.8–2.2	3.4–5.9	1.2–2.2	1.4–1.8	1.2–1.7

## Data Availability

The original DICOM images used in this study can be provided on reasonable request.
